# Bridging language and data: Transforming agricultural curricula for data analytics through linguistic insights

**DOI:** 10.1371/journal.pone.0348935

**Published:** 2026-05-27

**Authors:** Zhihong Xu, Jaehyun Ahn, Shuai Ma, Anjorin Ezekiel Adyemi, Fahmida Husain Choudhury, Xiting Zhuang, Rafael Landaverde, Gary Wingenbach

**Affiliations:** 1 Department of Agricultural Leadership, Education, and Communications, College Station, Texas A&M University, TX, USA; 2 University of Florida, Gainesville, FL, USA; 3 Fort Hays State University, Hays, KS, USA; 4 North Dakota State University, Fargo, ND, USA; PNG National Research Institute, PAPUA NEW GUINEA

## Abstract

In response to the growing demand for data analytics competencies in Food, Agriculture, Natural Resources, and Human (FANH) Sciences, this study investigated how linguistic and demographic differences among learners can inform differentiated curriculum design. The objective was to identify distinct learner subgroups and explore how their expressed needs, tool preferences, and curricular priorities vary, thereby guiding the development of inclusive and responsive data analytics programs. Using a mixed-methods approach, the research team surveyed 535 alumni from a land-grant university, collecting both quantitative and qualitative data. Clustering analysis revealed two distinct groups: younger professionals emphasizing technical proficiency (e.g., coding, visualization, tool fluency), and older professionals prioritizing strategic competencies (e.g., leadership, communication, conceptual reasoning). Text mining of open-ended responses further highlighted divergent word usage patterns across curriculum dimensions, such as background tools, core topics, and supplemental skills, which validated the cluster distinctions. Key findings show that Group 1 (i.e., younger respondents with less work experience) favored hands-on, tool-centric learning, while Group 0 (i.e., older respondents with less education but with more experience) emphasized integrative applications and strategic thinking. These insights suggest that a one-size-fits-all curriculum is insufficient in curriculum design and development. Instead, differentiated learning pathways such as technical labs for early-career learners and strategic modules for experienced professionals are essential to accommodate learners’ needs. Communication skills emerged as a critical bridge across groups, underscoring the need to embed interpretive and collaborative competencies alongside technical training. This study demonstrates the value of combining linguistic analysis with demographic clustering to inform curriculum development. By aligning educational offerings with learner profiles and industry expectations, FANH science programs can better prepare graduates for diverse roles in data-driven agricultural and environmental science sectors.

## Introduction

In the context of digital transformation and the rising demand for analytical skills, the development and implementation of a comprehensive data analytics curriculum have become both a necessary and competitive academic offering for higher education students in the disciplines of Food, Agriculture, Natural Resources, and Human (FANH) Sciences [1,2]. These disciplines, central to food security, economic development, and the sustainability of agrifood systems, require professionals who can collect, analyze, and interpret complex data to support informed decision-making and foster innovative solutions to contemporary productive and environmental challenges. As Bounaris et al. (2022) [1] note, most students in FANH science-related academic programs recognize the importance of preparing in data analytics to remain competitive in today’s labor market.

As in many other disciplinary areas, teaching data analytics within the FANH sciences presents challenges due to the diversity of student profiles, including their prior experiences and, more critically, their learning needs [3,4]. Educational researchers emphasize that heterogeneity of students enrolled in the FANH sciences need a curriculum that is flexible, adaptive, and responsive to the cultural, academic, and professional contexts of learners [5]. In cultivating data analytics competencies, educators must account for varying levels of exposure to technology, proficiency in statistical techniques, and familiarity with digital environments, all of which can influence the effectiveness of teaching and learning [6,7]. According to Daum (2025) [8], achieving mastery in data analytics requires learners to acquire theoretical knowledge and build practical skills through hands-on experience, ultimately becoming competent and confident professionals.

In the United States, there is growing multisectoral interest in preparing agricultural professionals who can meet the demands of a rapidly evolving and unpredictable production landscape. Many current educational investment initiatives in the FANH sciences aim to address challenges similar to those that inspired this study. This study is grounded in the premise that understanding differences among learner subgroups provides valuable insights for curriculum development, planning, and implementation in data analytics education [9], and through the analysis of linguistic patterns, tool preferences, proposed course topics, and desired skills, this article seeks to explore how perspectives and learning needs vary across different demographic and experiential profiles.

Our study was guided by the following research questions: RQ 1: Are there distinct subgroups of users based on their demographic and experiential profiles that may inform curriculum needs in data analytics education in the FANH sciences? RQ2: If distinct subgroups exist, how do their expressed needs and perspectives differ in ways relevant to curriculum design? Specifically, 1) How do overall word usage patterns differ between Groups, revealing divergent emphases or priorities, and 2) How do groups differ in their preferences for tools and learning resources, proposed course topics and activities, and additional skills, particularly in relation to designing a data analytics curriculum in the FANH sciences tailored to a varied learner profile?

## Literature review

### Advancing data analytics curriculum in the FANH sciences

FANH science graduates play crucial roles in redefining agricultural science, developing technology, and managing natural resources. Their contributions are vital in addressing food security, nutrition, and international food trade, emphasizing the interconnectedness of food, agriculture, and humanity [10-13]. Graduates and professionals in the FANH science sector are considered strategic national assets and are essential for maintaining food availability, access, utilization, and stability, all of which are foundational to national prosperity and resilience [14]. Goecker et al. (2015) [15] reported more than 50,000 annual job openings for college graduates in food, agriculture, renewable natural resources, and environmental sciences. Furthermore, there is a growing appreciation of the importance of expanding student participation in these sciences by advancing educational opportunities, particularly for underrepresented groups [16].

However, developing this robust workforce in the FANH sciences requires multisectoral collaboration through enhanced education and training programs at both institutional and governmental levels [11,12]. With rapid technological advancements, students in the FANH sciences need more practical and up-to-date skills to meet the evolving demands of industry and society effectively. According to Gabriel and Aluko (2019) [17], skill-based training is crucial for responding to societal and industrial needs, as it bridges the gap between knowledge and actionable competence. Supporting this, Maisiri et al. (2019) [18] noted that the current wave of Industry 4.0 is reshaping all sectors, including those within the FANH sciences. They further emphasized that this shift is driven by the incorporation of advanced technologies such as automation and data analytics.

Additionally, the application of data science and machine learning skills is increasingly used in agriculture to make farming tasks more accurate and efficient. McLeod et al. (2017) [19] illustrated the benefits of data analytics in agriculture, explaining that it enables farmers to track crop yields, forecast results, and enhance resource use. They also stressed that incorporating robust data analytics education equips future professionals to harness large datasets for higher crop productivity, sustainable resource management, and evidence-based approaches in human sciences—contributions that ultimately enhance resilience and effectiveness across the sector, a perspective also endorsed by Chergui et al. (2020) [20].

Based on the aforementioned research findings, employers today seek graduates who demonstrate not only theoretical subject-matter expertise but also the ability to apply their knowledge in practical settings. Almgerbi et al. (2021) [21] noted that systematic mapping of job skills against educational offerings ensures relevance and employability. Moreover, while many advocate for integrating data analytics into agriculture education, Weissgerber (2021) [22] emphasized the need for realistic and field-specific training, cautioning that students may lack adequate preparation to fully engage with complex data science tools. She noted that without proper support, this shift could lead students to rely on familiar but outdated techniques. This concern is similarly shared by Nychas et al. (2021)[23], who added that it might shift attention away from hands-on practices critical to food safety and agricultural success.

Almgerbi et al. (2022) [21] noted a clear gap between expected skills and what is offered in data analytics courses, stating that a systematic mapping of job postings and online courses reveals that while demand for data analytics expertise is high, educational offerings often lag behind the analytics skills required by employers. Therefore, modern curricula must emphasize not just technical proficiency but also adaptability to technological disruptions. A robust data analytics curriculum is therefore critically needed in the fields of food, agriculture, natural resources, and human sciences to equip graduates with the skills necessary to address complex, data-driven challenges. Tapis and Priya (2020) [24] added that data analytics curricula help bridge gaps between disciplines by providing common analytical frameworks and fostering collaboration among food scientists, agronomists, environmentalists, and human scientists.

To address this gap, educational institutions increasingly recognize the need to update curricula and provide capability development frameworks that enhance students’ creativity, critical thinking, problem-solving, digital literacy, and data analytics skills [25]. For example, the EVAL Framework was developed to enhance evaluation competencies and leadership skills among FANH science graduates through experiential learning, mentoring, and project-based activities [26]. However, challenges remain, including inadequate educational infrastructure and institutional resistance. To respond, universities are forming partnerships with software vendors and industry alliances [19], offering hands-on experiences with real-world datasets and analytics tools to prepare students for data-centric roles [19,24]. Integrating practitioner involvement and adaptability measures ensures curricula remain responsive to student needs and industry expectations [24].

### Tailoring curriculum design to diverse student needs

The accelerating integration of data science and machine learning into agriculture, food, and natural sciences reflects a broader transformation in how knowledge is produced and applied within these domains. Employers across agribusiness, food systems, environmental management, and natural resource industries increasingly demand graduates who are not only proficient in disciplinary theory but also capable of leveraging data analytics tools to address complex, data-driven challenges. Precision agriculture, supply chain optimization, climate modeling, and sustainable resource management all depend on the ability to interpret large and heterogeneous datasets, develop predictive models, and translate computational outputs into actionable decisions [27,28]. Consequently, higher education must align curriculum design with workforce expectations, embedding data analytics competencies into disciplinary contexts to produce graduates who are adaptable, technologically equipped, and industry-ready [1,29].

At the same time, the diversity of student populations entering programs in the FANH sciences highlights the need for inclusive curriculum design. Learners come with varying levels of exposure to mathematics, statistics, and computational thinking, as well as differing cultural and professional experiences that shape how they engage with data analytics concepts [30-32]. A uniform, technically intensive curriculum risks excluding students without prior quantitative training, while overly simplified approaches fail to equip graduates for the demands of modern agricultural and environmental sectors. Curriculum innovation must therefore balance rigor with accessibility, creating scaffolded pathways that allow students from different backgrounds to progressively build data analytics expertise.

The increasing need to make curriculum design cater to the increasing diversity of the student populations across the globe due to globalization is a subject that has been well researched [33-35]. Too often, course content struggles to align with the varied backgrounds, experiences, and learning preferences of students [36]. Oladejo (2005) [37,38], emphasized that incorporating culturally responsive teaching practices validates student identities and promotes empathy, respect, and belonging among learners. He also said that tailoring curriculum design to diverse student profiles enhances the effectiveness of teaching by addressing individual learning needs. This is supported by some researchers such as Hao (2024) [39] and Schwenger (2024) [40] have however offered critical perspectives, noting that frequent curriculum adjustments to accommodate diversity may undermine consistency and stability in educational programs. They however also added that tailoring curricula to diverse learners can significantly increase demands on educator resources, including time, support, and specialized skills. Nonetheless, the prevailing view is that curriculum designers must move beyond one-size-fits-all approaches. Student diversity, including variations in cultural background, language, prior knowledge, and cognitive ability, should be the norm, not the exception. Failing to consider this often leads to ineffective learning environments and the need for costly retroactive accommodations [41]. Instead, integrating learning analytics with personalized learning design is seen as key to ensuring inclusive education for all learners.

Furthermore, research indicates that student background factors, such as prior grades and coursework, as well as involvement in the course (e.g., attendance, participation), predict academic achievement [42]. Law and Liang (2020) [36] emphasized that tight coupling between learning outcomes, task sequence design, and analytics/feedback is essential when analytics and feedback are integrated from the outset, rather than added later, course design becomes more responsive to student needs. Blumenstein (2020) [43] noted that students often enter courses with different levels of prior knowledge, making a single-level course either too advanced or too basic for many. Additionally, some courses may fail to reflect current industry practices and tools, making them less relevant to students aiming for practical or advanced skills [36,43].

The shift from traditional to digital and blended learning environments introduces further complexity. Rigid delivery formats may not suit the diverse situations and learning styles of modern students [44]. Hence, incorporating diversity into curriculum design helps bridge the gap between intended learning outcomes and what students actually achieve, leading to more inclusive and effective education [45].

Finally, developing a dynamic and inclusive curriculum requires systematic needs analysis using interviews, surveys, and classroom observations. Triangulating these approaches allows educators to uncover both explicit and implicit needs, ensuring curricula respond to students’ lived experiences and aspirations. According to Aghnia et al. (2025) [46], teachers are urged to act as ‘curriculum detectives’, not only carrying out the curriculum but also critically examining and adjusting it with attention to data and sensitivity to students’ experiences.

## Methods

### Participants and data collection

To develop a data analytics curriculum tailored to different users’ needs, the research team at a state flagship land-grant university obtained contact information for 13,962 alums from the College of Agriculture and Life Sciences. All procedures involving human participants were conducted in accordance with international ethical standards and the principles of the Declaration of Helsinki. Participants were provided with information about the purpose of the study, study procedures, potential risks and benefits, and their voluntary right to participate and withdraw at any time without penalty. Written informed consent was presented at the beginning of the Qualtrics survey, and participants indicated consent by selecting “I agree” to proceed. No minors were involved in the study. The study protocol was reviewed and approved by the Institutional Review Board (IRB) at Texas A&M University (IRB Number: IRB2023-0899M).

The team distributed a voluntary Qualtrics survey that included both quantitative and qualitative questions. As an incentive, a $10 gift card was offered to the first 300 respondents. A total of 636 survey responses were collected. After data cleaning, 535 valid responses were retained for analysis. These responses provided essential mixed-methods data to inform the curriculum design.

### Instrument

The researchers developed a mixed-methods survey instrument consisting of both closed- and open-ended items to assess respondents’ demographic characteristics, data analytics knowledge and skills, and professional practices.

The demographic section captured key variables such as educational level, gender, age group, years of work experience, and current workplace usage of data analytics. These variables provided the foundation for clustering respondents by background and experience level in the prerequisite analysis. For instance, nearly half of the respondents (49%) reported 0–5 years of professional experience, and a majority (63%) indicated that they currently use data skills in their workplaces. The disciplinary background was also considered, with over half the participants majoring in Agricultural or Environmental Sciences. The survey revealed a broad range of academic and professional diversity– most respondents held bachelor’s degrees, and female alumni comprised a larger portion of the sample (See Table 1 for summary statistics).

**Table 1:** Background information (n = 535).

Disciplinary focus			
Code	Categories	n	%
1	Agricultural/ Environmental Science	316	59
2	Life Science/ Biotechnology	123	23
3	Social Sciences/ Education	68	13
4	Hospitality/ Management	28	5
Educational Level			
Code	Categories	n	%
1	Bachelor	286	53
2	Master	159	30
3	Doctorate	84	16
4	Other	6	1
Gender			
Code	Categories	n	%
1	Male	192	36
2	Female	335	63
3	Prefer not to say	8	1
Age			
Code	Categories	n	%
1	22-27	224	42
2	28-33	254	48
3	34-39	33	6
4	40 and above	24	4
Work Experience in Year			
Code	Categories	n	%
1	0-5	263	49
2	6-10	195	36
3	11-15	47	9
4	More than 16	30	6
Usage (Big Data Analytics Skills)			
Code	Categories	n	%
0	Non-use	198	37
1	Use	337	63

The second section of the survey assessed eight core knowledge and skill domains, adapted from established literature [47,48, Jones, 2020, 49]. These domains included: basic concepts of data analytics (e.g., coding, modeling, data cleaning), data management and security (e.g., encryption, firewall management), data visualization (e.g., graphs, dashboards), statistical techniques (e.g., descriptive statistics, correlation, t-tests), higher-order analytical skills (e.g., advanced modeling, prediction models, factor analysis), machine learning (e.g., social network analysis, natural language processing), critical thinking (e.g., problem-solving, decision-making, self-reflection), communication skills (e.g., collaboration, teamwork, presentations). Participants rated each item using a 5-point scale ranging from 1 (Strongly Disagree) to 5 (Strongly Agree), providing data on both perceived proficiency and training needs.

To gather deeper insights, the survey also included three open-ended questions addressing participants’ workplace tools, learning resources, course design suggestions, and additional skills needed for career success:

- Item1: What specific data analytics and visualization tools do you use in your current job? Since leaving [a state’s flagship land-grant institution], what online programs have you used to learn about data analytics and visualization?
- Item 2: If you were creating an academic course for data analytics, what topics and activities would you include to help prepare the student in the curriculum for entry-level positions?
- Item 3: What additional knowledge and skills, other than data analytics or visualization, are needed for success in your profession?

These qualitative responses provided contextual understanding of alumni expectations and emerging trends, contributing directly to curriculum design.

### Data analysis

The data analysis was conducted in two phases, integrating both quantitative clustering and qualitative text mining to explore group differences and inform curriculum design.

Clustering validation using the silhouette score and Calinski–Harabasz index supported a two-cluster solution (k = 2). We subsequently labeled the two respondent groups as Group 0 and Group 1. We examined open-ended responses by prompt category (Background, Core/Role, Supplemental), comparing word usage within each category between the two groups (B0 vs. B1, K0 vs. K1, S0 vs. S1).

### Phase 1: Clustering analysis

We first used machine clustering techniques in Python to identify distinct subgroups based on selected demographics features. The goal was to group participants with similar backgrounds to better understand differences in their responses. Two primary clusters emerged: one smaller group (n = 96) and one much larger group (n = 439), reflecting uneven sample sizes.

### Phase 2: Text mining and linguistic comparison

Next, we analyzed open-ended survey responses using text mining methods in RStudio to compare linguistic patterns between groups. The responses were categorized according to three core dimensions: Item 1 focused on participants’ background, tools, and resources, labelled as B0 and B1; item 2 addressed content related to the core curriculum, categorized as K0 and K1; item 3 offered supplementary insights to support curriculum development, classified as S0 and S1.

To ensure fair comparisons between groups with unequal sizes, we normalized all word counts using relative frequency. Rather than comparing raw word counts– which would be biased toward the larger group– we divide each word’s count by the total number of words in its respective group. This adjustment allowed us to observe proportional word usage independent of group size, making comparisons more meaningful and statically valid.

We treated text preprocessing and tokenization carefully. Open-ended responses were analyzed in R using a tidytext workflow. Text was lowercased and tokenized into unigrams. Standard English stopwords were removed using the tidytext stopword lexicon. We did not apply stemming or lemmatization to preserve interpretable, curriculum-relevant terms. To reduce fragmentation due to formatting and spelling variants, we normalized a small set of common expressions before analysis; for example, “Power BI” and “PowerBI” were harmonized to a single canonical form for counting. For reporting consistency, a small set of near-equivalent terms was also harmonized (e.g., “analysis” → “analytics”). Word usage was summarized as relative frequency (token count divided by total tokens) within each group/subgroup to support comparability across groups with different response volumes.

We used several visualization techniques to explore word usage. To provide an initial overview of word usage, the team generated bar charts using the *ggplot2* package in R. These charts displayed the top words by relative frequency for each group. We also employed word clouds to provide the most salient terms, with word size representing proportional usage within each group. The *wordcloud* and *wordcloud2* packages facilitated these visualizations.

To maintain clarity, we pre-filtered the text data to include only the top 20–50 most frequent words per group using *dplyr* (e.g., slice_max(n, n = 50)). This step helped focus on the most relevant content and avoided overwhelming the visuals with frequent terms.

For deeper comparison, we organized the data into a matrix (with words as rows and groups as columns). Using the pheatmap package, we generated heatmaps to visually compare word usage patterns across all subgroups (e.g., B0/B1, K0/K1, S0/S1). Each row was standardized (z-score scaling) to represent words that appeared significantly more or less frequently in a given group. Hierarchical clustering further revealed patterns in how words and groups aligned based on usage similarity.

This structured workflow– data preparation (importing and normalizing); visual overviews (bar charts and word clouds); in-depth comparisons (heatmaps with hierarchical clustering) – enabled us to uncover both broad trends and nuanced differences in participant responses. The resulting visuals and comparisons provided valuable insights into group-specific priorities, tools, and expectations– informing the curriculum development with both quantitative and qualitative depth.

## Results

### Cluster analysis

We identified two distinct groups based on selected linguistic and demographic features using unsupervised machine learning techniques in Python. To determine the optimal number of clusters, we relied on internal validation metrics– specifically the Silhouette Score and the Calinski–Harabasz Index– both widely used to assess clustering quality. The silhouette score indicates how well each data point fits within its cluster, balancing cohesion and separation. The Calinski-Harabasz score evaluates cluster compactness and the degree of separation between them, with higher values indicating better defined clustering structures.

**Fig 1:**
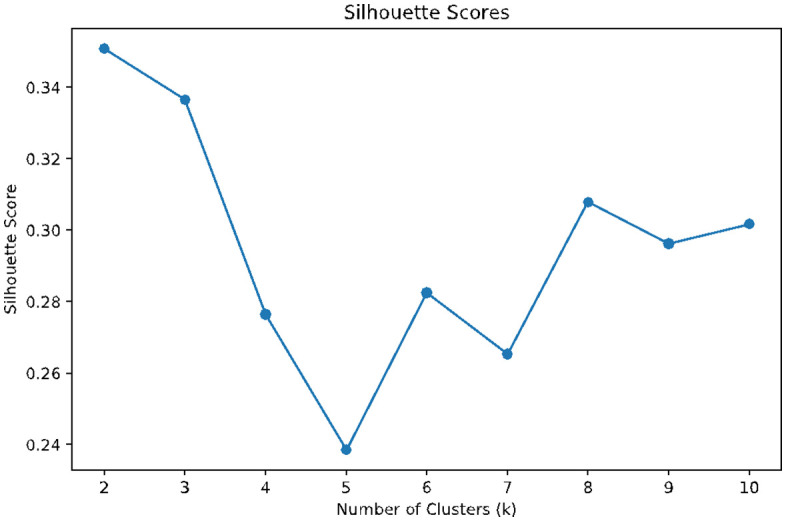
Silhouette scores. The figure displays average Silhouette Scores for cluster solutions ranging from k = 2 to k = 10. Higher Silhouette Scores indicate better-defined clusters with greater cohesion and separation. The score reaches its maximum at k = 2, supporting the selection of a two-cluster solution.

**Fig 2:**
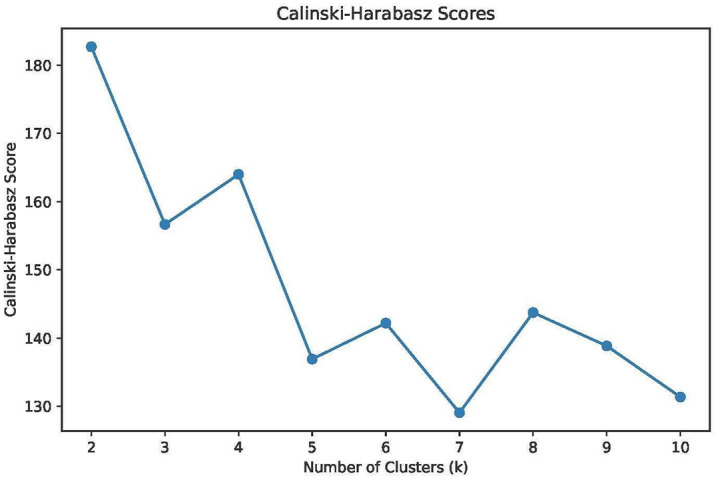
Calinski–Harabasz scores. This figure presents Calinski–Harabasz index values for cluster solutions with k = 2 to k = 10. Higher values indicate a more favorable balance between within-cluster similarity and between-cluster separation. The index is maximized at k = 2, indicating that this solution provides the strongest overall clustering structure.

As shown in Figs 1 and 2, both metrics pointed to two clusters (k = 2) as the optimal solution. The Silhouette Score peaked at k = 2, indicating the strongest overall fit between data points and their respective clusters. Similarly, the Calinski-Harabasz index showed a notable maximum at k = 2, suggesting that this solution offered the best tradeoff between intra-cluster similarity and inter-cluster distinction. Thus, selecting two clusters allowed us to retain a high degree of interpretability while preserving meaningful group differences.

**Fig 3:**
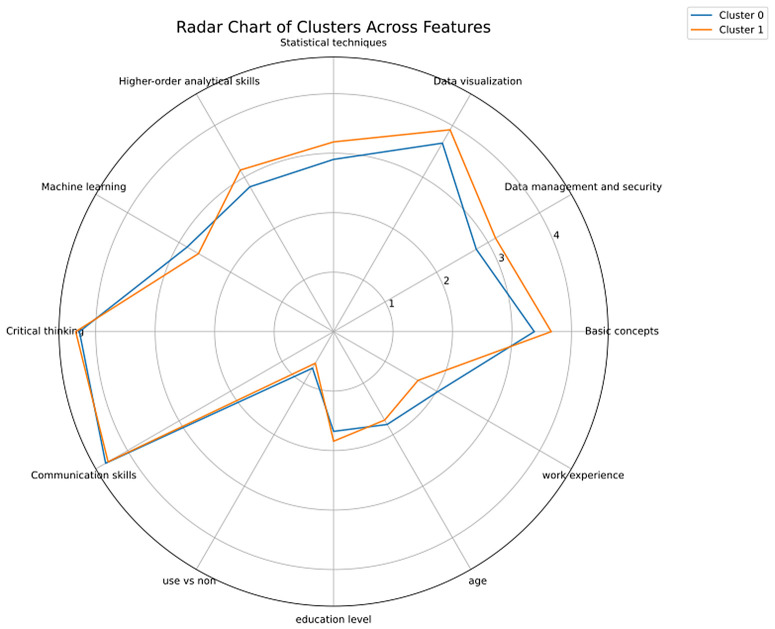
Radar chart of cluster characteristics.

To further interpret the clusters, we used a radar chart (Fig 3) to visualize the average values of key features of each group. Results of analyzing descriptive statistics of two clusters indicated that Cluster/group 0 is older, with lower educational level, more work experience, and placed higher value on advanced tools such as big data analytics and machine learning. Cluster/group 1 includes younger respondents with less work experience, who prioritized foundational concepts including basic data concepts, infrastructure, data management and security, and data visualization. This two-cluster solution not only reflects statistically valid groupings but also reveals meaningful pedagogical insights for differentiated curriculum design.

### Textual analysis

We next analyzed responses to three open-ended survey items, linking each respondent’s qualitative input to their assigned cluster (Group 0 or Group 1). Group 0 primarily included more experienced professionals with longer work histories, while Group 1 comprised younger respondents with less professional experience, likely earlier in their careers. This allowed us to explore linguistic patterns across curriculum dimensions– Background (B, corresponding to Item 1 questions), Core Curriculum (K, corresponding to Item 2 questions), and Supplemental (S, corresponding to Item 3 questions) – using text mining techniques including word frequency analysis, word clouds, and heatmaps.

### Overall word usage patterns differ between two groups

Table 2 and 3 present the top 20 words with the largest positive and negative shifts in relative frequency from Group 0 to Group 1 across all open-ended responses. Table 2 lists the words that show the most substantial positive shifts in Group 1, reflecting an emphasis on the application of tools and workflow execution. Several of the most notable increases are software- and practice-oriented terms, such as *Excel*, *visualization*, *tools*, *Python*, and *skills* (alongside related terms like cleaning, code, and programming, where applicable). For example, the term Excel increases from approximately 1.36% of all tokens in Group 0 to 2.35% in Group 1 (difference ≈ 0.99 percentage points), indicating stronger attention to tool fluency and applied practice among Group 1 respondents. The term “*analytics*” is commonly used in both groups. In Group 1, its usage shows a modest increase, indicating that what sets this group apart is not just the value placed on analytics, but rather the specific tools and procedural skills employed to implement them.

**Table 2:** Top 20 positive shifts (Group 0 → Group 1).

Word	Relative frequency Group 1	Relative frequency Group 0	Difference
Excel	0.02352	0.01362	0.00989
Visualization	0.00852	0.00409	0.00443
Real	0.00716	0.00272	0.00443
Tools	0.00784	0.00409	0.00375
Skills	0.00750	0.00409	0.00341
Management	0.00341	0.00000	0.00341
Analytics	0.03170	0.02861	0.00309
Python	0.00579	0.00272	0.00307
Cleaning	0.00307	0.00000	0.00307
Activities	0.00409	0.00136	0.00273
Code	0.00273	0.00000	0.00273
Examples	0.00273	0.00000	0.00273
Introduction	0.00273	0.00000	0.00273
Learn	0.00273	0.00000	0.00273
Statistics	0.01057	0.00817	0.00239
Project	0.00375	0.00136	0.00239
Importance	0.00239	0.00000	0.00239
Microsoft	0.00239	0.00000	0.00239
Multiple	0.00239	0.00000	0.00239
Programming	0.00239	0.00000	0.00239

**Table 3:** Bottom 20 negative shifts (Group 0 → Group 1).

Word	Relative frequency Group 1	Relative frequency Group 0	Difference
Findings	0.00068	0.00409	−0.00341
Marketing	0.00068	0.00409	−0.00341
Story	0.00068	0.00409	−0.00341
Critical	0.00307	0.00681	−0.00374
Knowing	0.00170	0.00545	−0.00375
Effectively	0.00034	0.00409	−0.00375
Patterns	0.00034	0.00409	−0.00375
Platforms	0.00136	0.00545	−0.00409
Related	0.00136	0.00545	−0.00409
Foundations	0.00000	0.00409	−0.00409
Roles	0.00000	0.00409	−0.00409
Specific	0.00204	0.00681	−0.00477
Formulas	0.00068	0.00545	−0.00477
Information	0.00307	0.00817	−0.00511
Company	0.00034	0.00681	−0.00647
Business	0.00273	0.00954	−0.00681
Basic	0.02284	0.03134	−0.00850
Training	0.00000	0.00954	−0.00954
Curriculum	0.00000	0.01090	−0.01090
Icon	0.00000	0.01090	−0.01090

In contrast, Table 3 presents the top 20 words with the most significant negative shifts from Group 0 to Group 1, indicating terms that they are more characteristic of Group 0. These terms are more conceptual or integration-oriented, including *curriculum*, *training*, *business*, *company*, *platforms*, *roles*, and *foundations*, reflecting a stronger focus on contextualizing analytics within organizational strategy and decision-making. This orientation consists of the emphasis of more experienced respondents on how analytics supports broader workflows and institutional objectives. For instance, one participant referenced “ITIL Best Practices to understand the value of the analytics to apply to business strategy and vision,” which illustrates the integration-focused framing typical of Group 0.

Overall, Tables 2, 3 show a clear distinction in emphasis: Group 1 more often references tool- and workflow-based competencies, whereas Group 0 more often references conceptual framing and organizational integration. This distinction in word usage aligns with the two-cluster structure identified in the quantitative clustering analysis. It supports treating the groups separately in subsequent text-based comparisons by prompt category (B0 vs. B1, K0 vs. K1, S0 vs. S1). Together, the clustering and text-mining results provide a robust foundation for tailoring curriculum design to differing priorities, experience levels, and learning needs across the two groups. The shifted terms suggest two complementary instructional emphases. Words increasing in Group 1 (e.g., Excel, visualization, tools, Python) indicate demand for hands-on tool fluency and applied workflow practice. In contrast, terms more frequent in Group 0 (e.g., curriculum, training, business, information) emphasize conceptual framing and application context. These patterns support a differentiated curriculum that pairs applied lab modules for tool-based competency development with strategy- and integration-oriented modules that connect analytics to organizational decision-making.

### Differences in subgroups

Building on the overall distinction between Group 0 and Group 1, we examined how this clustering manifests within three key curriculum dimensions: Background (B), Core Curriculum (K), and Supplemental (S). The groups were identified through clustering based on respondents’ self-assessed proficiency ratings across eight data analytics skill domains measured on Likert-scale survey data. After clustering, we examined demographic characteristics to interpret the groups. As shown in Table 4, both groups exhibited similar age distributions, with the majority of respondents aged 22–33 years (Group 0: 88%; Group 1: 89%). However, the groups differed notably in work experience: Group 0 consisted of more experienced professionals, with 68% having 6 + years of experience, while Group 1 comprised earlier-career respondents, with 53% having 0–5 years of experience. Education levels were comparable across groups, with approximately half holding bachelor’s degrees (Group 0: 55%; Group 1: 53%).

**Table 4:** Descriptive statistics by cluster.

Demographics:	Cluster 0 (n = 96)	Cluster 1 (n = 439)
**Age group**		
**22-27 years**	34 (35%)	190 (43%)
**28-33 years**	51 (53%)	203 (46%)
**34-39 years**	7 (7%)	26 (6%)
**40 + years**	4 (4%)	20 (5%)
**Work experience**		
**0-5 years**	30 (31%)	233 (53%)
**6-10 years**	44 (46%)	151 (34%)
**11-15 years**	12 (12%)	35 (8%)
**16 + years**	10 (10%)	20 (5%)
**Education level**		
**Bachelor’s degree**	53 (55%)	233 (53%)
**Master’s degree**	33 (34%)	126 (29%)
**Doctoral degree**	0 (0%)	0 (0%)
**Other**	10 (10%)	80 (18%)
**Gender**		
**Male**	34 (35%)	158 (36%)
**Female**	62 (65%)	273 (62%)
**Prefer not to say**	0 (0%)	8 (2%)

#### Background (B): Tools and resources.

Open-ended responses to Item 1, “What specific data analytics and visualization tools do you use in your current job? What online programs have you used to learn about data analytics and visualization,” revealed distinct subgroup differences.

Across both groups, frequently mentioned tools include *Excel, Tableau, R, Power BI, Python, Graphs, SQL and SAS*, while common learning platforms include *Youtube, LinkedIn learning, Coursera, Google and Udemy*. These responses align with the quantitative likert-scale findings, which indicated high self-ratings in data visualizations skills across the sample [Bhatia, 2019].

**Fig 4:**
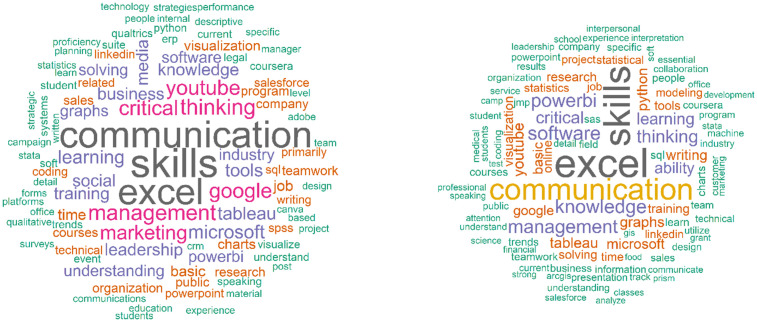
Word Cloud (B0 vs. B1). Note: The left side shows B0, while the right-side displays B1.

However, the word cloud Fig 4 and frequency table (Table 2) show sharper contrasts between the subgroups. B1 (Group 1) responses featured tool-specific terms such as *Excel*, *analytics*, and *PowerBI*, *Software*, *Python*, reflecting a technically focused orientation. In contrast, B0 (Group 0) responses emphasized broader competencies like *leadership, management, and skills*, pointing to a more strategic or supervisory perspective.

These patterns also appeared in the heatmap, where Group 1 columns in the Background dimension showed higher usage of specialized software terms, while Group 0 columns leaned toward general or managerial terminology.

Age-related differences also shaped platform preferences: older professionals (Group 0) more frequently mentioned YouTube, while younger professionals (Group 1) cited tools like Power BI more often, suggesting differences in preferred modes of upskilling.

#### Core curriculum (K): Topics and activities.

Participants were asked Item 2: “If you were creating an academic course for data analytics, what topics and activities would you include to help prepare the student in the curriculum for entry-level positions?”

**Fig 5:**
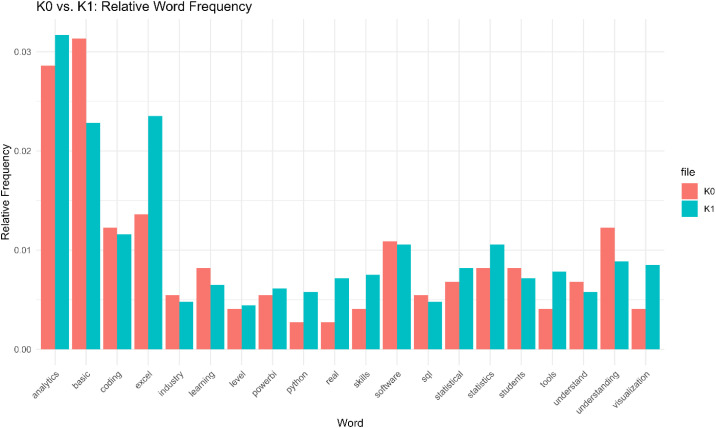
K0 vs. K1: Relative word frequency.

For both groups, commonly cited included *coding skills, data visu*alization, *tools (e.g., Excel, R, Power BI, Tableau, Python), statistical analysis, and data management (e.g., data organization, data cleaning)*. Yet, the relative frequency bar chart (Fig 5) and text patterns show that K1 (Group 1) emphasized terms such as *analytics, tools, statistics, and visualization*, consistent with a hands-on, tool-centric perspective.

Meanwhile, K0 (Group 0) responses gravitated toward foundational and conceptual terms like *coding and understanding*. This reflects a curriculum design preference rooted more in principles and frameworks– consistent with their older age and experience.

#### Supplemental (S): Additional knowledge and skills.

In response to Item 3 “What additional knowledge and skills, other than data analytics or visualization, are needed for success in your profession?” Both groups commonly mentioned *communication, critical thinking, and problem solving.*

**Fig 6:**
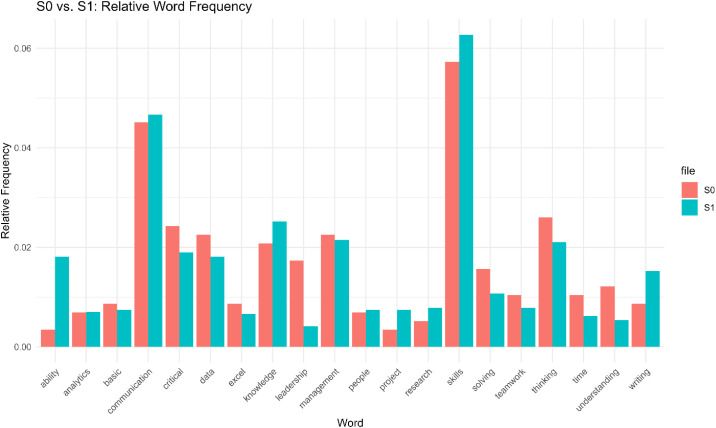
S0 vs. S1: Relative word frequency.

However, the bar chart in Fig 6 reveals that S1 (Group 1) respondents placed higher emphasis on technical competencies like *research, data, Excel, and writing,* reinforcing the pattern observed in earlier dimensions. S0 (Group 0), on the other hand, highlighted interpersonal and managerial skills such as *communication*, *teamwork*, *understanding, and leadership.*

Quotes from Group 0 participants reinforce these findings: *“It is really crucial to learn how to communicate effectively with a non-technical audience. Students should be trained to interpret model results or statistics in layman’s terms and to avoid fixating their preference on using technical jargons unconsciously.”* These reflections suggest that while Group 1 emphasizes technical execution, Group 0 underscores the importance of strategy, communication, and decision-making– a pattern closely tied to age and role differences.

#### Heatmap synthesis.

**Fig 7:**
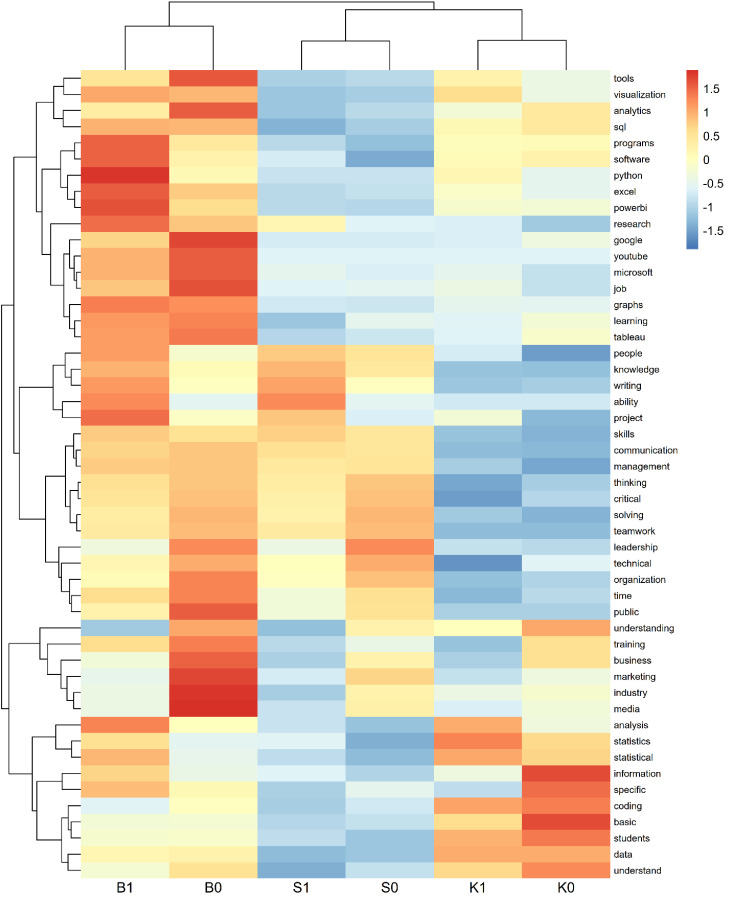
Heatmap of B, K, S.

The heatmap visualization (Fig 7) consolidates text mining results across the Background (B), Core Curriculum (K), and Supplemental (S) dimensions and reinforces group-level distinctions. Within each curriculum dimension, Group 1 consistently shows higher frequency of tool-specific and analytic terms, while Group 0 reflects stronger usage of conceptual, managerial, or people-oriented language. The difference between Group 1 and Group 0 is closely tied to age and professional experience.

By combining quantitative survey clustering with open-ended text mining, we find that age and experience meaningfully shape curricular priorities. Younger, less experienced professionals (Group 1) gravitate toward specialized tools and technical skills, while older professionals (Group 0) emphasize strategic thinking, leadership, and communication. These findings suggest that curriculum designers should offer differentiated learning pathways that cater to diverse professional profiles and career stages.

## Discussion

### Learner differentiation: Demographic and epistemic patterns

The identification of two distinct learner clusters based on linguistic and demographic features is not merely a methodological outcome. Rather, it points to the differentiated educational trajectories that professionals in FANH science-related data science may follow, trajectories that are often conditioned by their career stage. Besides, the internal validation through both the Silhouette and Calinski–Harabasz indices [50] demonstrated that *k* = 2 provided the strongest balance of cohesion and separation, reinforcing that two clusters were not an arbitrary partition but the most stable structure. The demographic composition of these clusters thus reflects professional stratification: older, experienced respondents (Group 0) focused on advanced tools framed in strategic contexts, while younger, less experienced respondents (Group 1) emphasized foundational technical skills.

This position aligns with the findings of Deming et al. (2019) [51], who observed that demographic attributes, particularly whether individuals inherited farmland, served as a significant determinant for Irish students pursuing careers in dairy farming. Such structural factors shaped not only their motivation to enter agriculture but also their broader educational experiences, which in turn influenced how they approached learning. Extending this to a much broader perspective, the emergence of these clusters also highlights the importance of recognizing heterogeneity in learner backgrounds when designing data science curricula for FANH science disciplines. Programs that account for demographic, cultural, and career-stage differences are better positioned to cultivate relevant skills and sustain learner engagement. Failure to do so risks reinforcing existing disparities, where individuals with specific advantages, such as land inheritance or prior exposure, may find educational pathways more accessible than those without such resources.

This duality aligns with Bloom’s taxonomy of learning [52,53], which conceptualizes learning as moving from lower-order cognitive processes (e.g., remembering, understanding and applying) to higher-order thinking processes (e.g., analyzing, evaluating and creating). In line with this framework, early-stage learners focus on foundational knowledge acquisition and skill application, while advanced learners emphasize higher-order competencies such as analysis, evaluation, and synthesis, consistent with findings from adult learning research [54-56]. For example, in this study, younger learners with less work experience perceived basic concepts, data management and security, and statistical techniques—which primarily involve remembering, understanding, and applying foundational knowledge—as more important. In contrast, older learners with more work experience placed greater importance on machine learning and communication skills, which involve higher-order cognitive processes such as evaluating and creating predictive systems, as well as synthesizing information and tailoring messages to diverse audiences.

In practical terms, the implication is that FANH science curricula must provide differentiated pathways. Novice learners should be guided through scaffolded modules that prioritize operational fluency with coding, visualization, and data cleaning [57,58]. More experienced learners should be engaged with integrative modules that situate advanced methods, such as machine learning or big data pipelines, within contexts of management, policy, and strategy [59]. Moreover, adult learning theory similarly posits that prior professional experience reshapes learning needs, with seasoned learners demanding relevance, problem-centered approaches, and immediate applicability [60-62]. The cluster analysis, therefore, validates stage-sensitive curriculum design grounded in developmental and adult learning principles.

Building on this demographic and developmental distinction, the lexical contrasts observed across groups further illustrate how learners frame their professional orientations and curricular priorities.

The contrasts in word usage also provide deeper insight into how learners frame their curricular priorities and professional pathways. Group 1’s vocabulary, centered on aspects such as *Excel*, *Python*, *programming*, *analysis*, and *visualization*, reflects a task-oriented approach [63,64] in which learning is viewed primarily as gaining technical tools for immediate application [65]. This aligns with ongoing calls to improve technical literacy in undergraduate FANH science programs, particularly in response to the shortage of entry-level professionals who can reliably clean, analyze, and visualize complex datasets. In contrast, Group 0’s repeated use of terms such as *curriculum*, *business*, *foundations*, *training*, and *roles* suggests a more integrative orientation, where the focus lies on connecting data with organizational structures, decision-making, and broader system-level applications [66].

This divergence points to a critical tension in curriculum design. A single, uniform model cannot adequately prepare learners when FANH science graduates are increasingly expected to operate in two distinct capacities: as technical specialists who can execute data-driven tasks and as integrators who can connect analysis to strategy in agricultural enterprises, food systems, and natural resource governance. The lexical evidence shows that learners themselves already recognize these differences, which suggests that curricula must be intentionally designed to reflect and cultivate both orientations. Otherwise, programs risk overemphasizing technical skills at the expense of broader integrative competencies, or vice versa, thereby producing graduates who are misaligned with the diverse and evolving demands of the FANH science workforce. For curriculum designers and policymakers, this therefore calls for a deliberate restructuring of programs toward a blended model that not only strengthens technical mastery but also embeds opportunities for integrative, decision-making, and leadership skills, ensuring that future professionals are fully equipped to meet the multifaceted challenges of the sector.

### Balancing technical proficiency and integrative competence

While learner trajectories differ, another clear theme is the balance between technical mastery and integrative application, revealed in both tool use and curricular emphasis.

### Tools and resources: Shared platforms, divergent orientations

The Background dimension also revealed a convergence in tool exposure across groups, with Excel, R, Python, SQL, and PowerBI forming a shared technological foundation. Yet, meaningful differences emerged in how these tools were understood and positioned within learning. Group 1 emphasized direct practice, approaching platforms as discrete objects of mastery and treating technical proficiency as the central goal. In contrast, Group 0 emphasized leadership and management functions, situating tools within broader organizational systems and framing them as instruments for decision-making and strategic oversight.

In FANH science contexts, these differences carry significant implications for both pedagogy and workforce preparation. In precision agriculture, for instance, early-career analysts may prioritize mastering the operation of drones, sensors, or farm management software, while experienced agronomists or extension specialists focus on interpreting these outputs to inform policy, resource allocation, or technology adoption decisions. Similarly, in food supply chain management, novice professionals may concentrate on developing SQL queries and interactive dashboards, whereas managers emphasize applying these outputs to contract negotiation, risk mitigation, or regulatory compliance. These contrasts illustrate that while the technological ecosystem is shared, the framing of tools as either objects of technical practice or components of organizational strategy diverge sharply by professional stage.

From a critical perspective, this finding challenges the assumption that exposure to common tools alone is sufficient preparation for FANH science careers. Research on agricultural workforce readiness has consistently highlighted mismatches between the skills emphasized in academic programs and the competencies required in practice. Studies such as Roberts and Edwards (2016) [67] and Lamm et al. (2023) [68] have documented persistent gaps between technical fluency and the ability to integrate data into managerial or leadership roles. Similarly, workforce development reports stress that technical proficiency must be complemented by systems thinking, communication, and leadership capacities if graduates are to thrive in rapidly evolving agricultural sectors [69]. Curricula that prioritize technical mastery as an end in itself risk neglecting the interpretive and managerial skills necessary for tools to be applied meaningfully. Conversely, those that focus predominantly on managerial application without sufficient grounding in technical practice risk producing graduates unable to engage critically with data.

Moreover, this tension is not unique to the FANH sciences. In engineering education, similar debates have emerged over the balance between technical rigor and managerial or design-oriented skills, with scholars arguing that graduates must be trained both as proficient problem-solvers and as innovators who can situate solutions in organizational and societal contexts [70]. Likewise, in public health education, programs often struggle to integrate biostatistical tool use with the leadership and policy competencies necessary to translate evidence into population-level interventions [71]. These parallels suggest that FANH sciences is part of a wider challenge across applied disciplines: the need to develop professionals who can both master tools and strategically deploy them in complex decision environments.

The evidence from this study, therefore, points to the need for differentiated yet complementary pedagogical approaches. Early stages of training should emphasize technical fluency and confidence with tools, while advanced stages should progressively situate those tools within decision-making, leadership, and governance contexts. Integrating these orientations within curricula would move beyond fragmented approaches and respond to calls for more holistic preparation of FANH science graduates who are not only capable of operating technologies, but also of transforming them into instruments of organizational innovation and sector-wide impact.

These differences in tool orientation extend into how learners view the very structure of the core curriculum, with one group prioritizing practical training and the other stressing conceptual reasoning.

### Core curriculum: Foundational practice versus conceptual reasoning

The Core Curriculum dimension revealed convergence around foundational competencies in *coding*, *visualization*, *statistics*, and *data management*, yet significant divergence emerged in emphasis and orientation. Group 1 respondents highlighted a practice-oriented curriculum reliant on hands-on technical skill acquisition. This orientation reflects an instrumental logic aligned with immediate employability, where demonstrable outputs such as scripts, dashboards, and models function as signals of job readiness. Group 0, by contrast, emphasized the need for conceptual understanding alongside coding, signaling a higher-order concern with frameworks, epistemic rationale, and principled application. Their perspective suggests that technical proficiency, in isolation, risks reducing education to training, producing technicians rather than reflective professionals capable of situating tools within methodological debates or ethical considerations.

This tension mirrors longstanding critiques in higher education between vocationalist and liberal traditions. As scholars have noted, the growing emphasis on job-ready skills responds to policy discourses on the skills gap and workforce alignment but often narrows the scope of education to short-term labor market demands [72,73]. It suggests that focusing on immediate applicability may obscure the cultivation of adaptive reasoning, critical literacy, and systems thinking, capacities essential for navigating complex, evolving FANH science challenges. Conversely, approaches that stress only interpretive or conceptual sophistication risk alienating learners seeking tangible skills for professional advancement, thereby reproducing inequities between those who can afford to pursue extended academic training and those who cannot.

In the FANH sciences, the practice–reasoning divide manifests in concrete ways. In environmental conservation, Group 1’s orientation translates into competence with geospatial analysis, coding pipelines for environmental data cleaning, and visualization of biodiversity indicators, skills indispensable for field-level practitioners tasked with rapid operational decisions. Group 0’s emphasis, however, foregrounds interpretive and integrative capacities such as using models to inform conservation policy, evaluating trade-offs in resource allocation, and synthesizing heterogeneous datasets into strategic recommendations. A similar distinction appears in agricultural education. Group 1 learners may prioritize applying R or Python to analyze student performance data, whereas Group 0 learners situate analytics within broader debates on curriculum reform, program evaluation, and educational policy.

Critically, framing these orientations as a binary outcome risks oversimplification. Literature on curriculum design argues that the false opposition between practice and reasoning undermines both dimensions, reinforcing the very divides it seeks to bridge [74,75]. Overemphasis on hands-on coding risks creating narrowly skilled graduates lacking adaptive versatility, while overemphasis on conceptual reasoning may leave learners ill-prepared for immediate labor demands. The pedagogical challenge, therefore, is not to privilege one dimension over the other but to scaffold their integration. Curriculum should be designed to enable translation between technical proficiency and higher-order interpretation, embedding opportunities for learners to move fluidly between tool use, critical reflection, and systemic application.

Such an integrated approach aligns with contemporary calls for critical vocationalism, where technical skills are situated within broader frameworks of knowledge, ethics, and social responsibility [76]. Within the FANH sciences, this means preparing learners not only to execute data analysis tasks but also to critically interrogate the assumptions underpinning datasets, the equity implications of analytic models, and the systemic consequences of their interpretations. By reconciling the immediate demands of workforce readiness with the longer-term imperatives of adaptive and critical reasoning, curricular design can resist reductive skills-gap framings and instead cultivate professionals equipped to lead within increasingly complex agricultural, environmental, and educational systems.

### Communication and supplemental skills as bridges

Beyond tools and curriculum development, supplemental skills, especially communication, emerged as a key area where orientations diverged but also where integration became possible. The Supplemental dimension contributes an important interpretive layer to the analysis of results by revealing differences in how groups conceptualize the role of transferable competencies. Both groups expressed value for communication, critical thinking, and problem solving, yet their emphases reflected distinct orientations. Group 1 situated supplemental skills within the axis of technical execution, linking them to writing, research, Excel proficiency, and data handling. These were viewed as auxiliary supports to the technical workflow, consistent with evidence that early-career professionals often equate skill development with technical proficiency. Group 0, however, framed supplemental skills as integrative competencies, emphasizing communication, teamwork, leadership, and interpretive capacity. In this view, such skills enable data science not merely to function as a technical process but to influence organizational decisions and broader systems [77].

The distinction carries significant implications for the FANH sciences. In agricultural extension, for instance, early-career professionals may prioritize producing written guides, technical manuals, or datasets intended for farmer use. By contrast, senior extension agents face the challenge of translating complex statistical outputs and model predictions into advice that is both accessible and actionable for stakeholders who lack technical expertise [78]. Similarly, within food systems, non-experienced analysts may concentrate on preparing consumer datasets and generating reports, while managers and decision-makers must synthesize these findings into strategic interventions for supply chains, market positioning, and policy compliance [79].

The quote from Group 0 emphasizing the translation of technical results into layman’s terms highlights an enduring tension in the pedagogical framing of data science within the FANH sciences: whether communication should be taught as an ancillary tool or as a foundational competency. Positioning communication as peripheral risks reinforces a technical–nontechnical divide, where students develop strong methodological skills but remain unable to bridge them into practice [80]. Conversely, embedding communication as a central, assessed outcome across curricula would align training with the demands of professional contexts where the ability to convey results persuasively and understandably often determines whether data-driven insights are adopted. The evidence suggests that curricula must avoid reproducing a hierarchy that singles technical capacity over integrative competencies, instead fostering a model where technical practice and communicative translation operate in tandem to achieve impact.

### Toward systemic curriculum models

Taken together, these findings reveal that the distinctions observed across clusters are not isolated but systematically patterned, pointing to the need for coherent program-level responses.

The heatmap analysis demonstrated that divergences across the Background, Core, and Supplemental dimensions were not incidental but systematically patterned. Group 1 consistently employed vocabulary associated with tool-specific proficiency and technical execution, whereas Group 0 favored language reflecting conceptual reasoning, managerial oversight, and integrative competencies. The recurrence of this polarity across dimensions validates the robustness of the two-cluster solution and suggests that the distinction is anchored in deeper epistemological orientations regarding the nature of professional competence [81].

Specifically, for curriculum designers, these findings indicate the necessity of systemic differentiation at the programmatic level rather than piecemeal modifications within individual courses. An execution-oriented pathway should integrate applied practice, laboratory-based activities, and iterative assessments that reinforce technical fluency, reflecting established principles of experiential and active learning [82]. Conversely, an integration-oriented pathway should emphasize leadership, governance, and strategic decision-making, consistent with scholarship highlighting the centrality of higher-order skills in complex professional environments [77]. Critical to this framework is the incorporation of bridging modules that maintain permeability between the two trajectories. Such modules may include translation projects, where novice learners rehearse the articulation of technical outputs for diverse audiences, and advanced learners periodically re-engage with emerging tools to sustain technical fluency. Designing these transitional elements prevents the creation of siloed tracks and instead produces professionals capable of moving fluidly between precision in execution and conceptual integration, a balance increasingly demanded in the FANH sciences [79,78].

## Conclusions, limitations, and future directions

In recent decades, higher education has increasingly emphasized career readiness, placing significant pressure on instructors across all fields to provide students with job-relevant skills that align with specific career paths [83]. This study contributes to FANH science sciences by bridging the gap between higher education training and industry workforce development. Data analytics is becoming a core competency in the FANH sciences, supporting decision-making in precision agriculture, food supply chain management, and environmental resource use. By identifying competencies and tools emphasized by students and alumni, educators can better align curricula with emerging demands.

The two clusters identified in this study confirm differentiated learner pathways. Younger professionals (Group 1) emphasize technical and data-intensive skills, while older professionals (Group 0) value technical foundations but also stress leadership, conceptual reasoning, and application. These findings parallel the discussion of demographic and epistemic orientations: Group 1 aligns with immediate technical fluency, and Group 0 with broader integrative competencies. Recognizing these differences enables curriculum designers to adopt differentiated strategies—advanced data labs for technically focused learners, and integrative projects for those pursuing strategic or managerial roles.

The study also demonstrates the importance of balancing technical proficiency with conceptual and integrative skills. While students and instructors often value core skills such as data analysis, management, and visualization [84], professionals with more experience highlight emerging techniques like machine learning as critical [85]. This does not diminish the role of foundational skills, but rather suggests the need for curricula that both reinforce technical literacy and situate advanced methods within organizational and strategic contexts. Integrating practice-oriented training with conceptual reasoning will ensure graduates are versatile and prepared for evolving industry demands.

Communication and supplemental skills further emerged as critical bridges. Both clusters recognized their importance, though younger learners linked them to technical execution, while experienced professionals emphasized communication, leadership, and translation for broader systems impact. Embedding these competencies across curricula ensures that graduates can conduct technical analyses and convey insights persuasively and accessibly, which is a demand echoed across FANH science professions.

Finally, the synthesis of results through clustering and text analysis underscores the need for systemic curriculum models. Isolated modifications are insufficient; instead, programs should design coherent pathways that integrate technical fluency, conceptual reasoning, and communication skills. Sequenced modules that build from foundational tools (B) to higher-order analytics (K), to supplemental skills (S) create clear learning trajectories and maximize synergy across courses [86].

This study has several limitations. First, the sample consisted of alumni with agricultural backgrounds from a single institution in one U.S. state, which may limit the generalizability of the findings. Second, the use of incentives may have attracted participants who are particularly motivated by compensation, potentially introducing selection bias. In addition, the reliance on self-reported measures of skills may not fully reflect participants’ actual competencies. The study also did not incorporate perspectives from industry employers, which limits insights into workforce expectations. Last, the cross-sectional design restricts data collection to a single time point and may not capture changes or long-term trends over time.

Future research can extend this methodological framework (i.e., surveys, clustering, and textual frequency analysis) beyond the FANH sciences to other STEM and interdisciplinary fields. Longitudinal studies could examine how skill priorities shift over time, and stronger integration of employer perspectives would further align curricula with workforce realities. By combining differentiated pathways, balanced skill development, and systemic integration, FANH science programs can future-proof their curricula and prepare graduates who are not only technically capable but also conceptually versatile, communicative, and ready to lead in a rapidly changing environment.
